# Interobserver reliability and diagnostic performance of Chiari II malformation measures in MR imaging—part 2

**DOI:** 10.1007/s00381-012-1763-3

**Published:** 2012-05-05

**Authors:** Niels Geerdink, Ton van der Vliet, Jan J. Rotteveel, Ton Feuth, Nel Roeleveld, Reinier A. Mullaart

**Affiliations:** 1 Department of Pediatric Neurology 801, Radboud University Nijmegen Medical Centre, P.O. Box 9101, 6500 HB Nijmegen, The Netherlands; 2 Department of Radiology, University Medical Centre Groningen, Groningen, The Netherlands; 3 Department of Epidemiology, Biostatistics and HTA, Radboud University Nijmegen Medical Centre, Nijmegen, The Netherlands

**Keywords:** Chiari II malformation, Spina bifida, MR imaging, Diagnostic performance

## Abstract

**Purpose:**

Brain MR imaging is essential in the assessment of Chiari II malformation in clinical and research settings concerning spina bifida. However, the interpretation of MR images of the malformation is not always straightforward. Morphometric analyses of the extent of Chiari II malformation may improve the assessment. In an attempt to select appropriate morphometric measures for this purpose, we investigated the interobserver reliability and diagnostic performance of several morphometric measures of Chiari II malformation on MR images.

**Methods:**

Brain MR images of 79 children [26 with open spinal dysraphism, 17 with closed spinal dysraphism, and 36 without spinal dysraphism; mean age 10.6 (SD 3.2; range, 6–16) years] were evaluated. All children had been assessed for Chiari II malformation (defined as cerebellar herniation in combination with open spinal dysraphism; *n* = 23). Three observers blindly and independently reviewed the MR images for 21 measures of the cerebellum, brainstem, and posterior fossa in three planes. The interobserver reliability was assessed by an agreement index (AI = 1 − RRE) and the diagnostic performance by receiver operating characteristic analyses.

**Results:**

Reliability was good for most measures, except for the degree of herniation of the vermis and tonsil. Most values differed statistically significantly between children with and without Chiari II malformation. The measures *mamillopontine distance* and *cerebellar width* showed excellent diagnostic performance.

**Conclusions:**

Morphometric measures may reliably quantify the morphological distortions of Chiari II malformation on MR images and provide additional tools to assess the severity of Chiari II malformation in clinical and research settings.

## Introduction

Chiari II malformation is a complex developmental malformation of the central nervous system. It is characterized by a small posterior fossa and downward displacement of the cerebellum and brainstem through an enlarged foramen magnum (hindbrain herniation) [1]. Chiari II malformation is almost uniquely associated with open spinal dysraphism [2]. McLone and Knepper [3] hypothesized that leakage of cerebrospinal fluid through the spinal anomaly reduces the distention of the embryonic ventricular system. The decreased inductive pressure on the surrounding mesenchyme results in an abnormally small posterior fossa. Approximately one third of the patients with Chiari II malformation develop signs and symptoms of brainstem compression [4]. The mortality in this symptomatic group is 15 to 35 % [5, 6]

Usually, Chiari II malformation is clinically diagnosed with help of MR imaging to assess severity. Although the malformation is characterized by a constellation of morphological features [7–11], the evaluation of MR images may not always be straightforward. A previous study showed that the assessment of several features is unreliable because judgment of these features varied between observers (see part 1). Assessment of MR images is complicated by the morphological diversity of the malformation, the qualitative nature of the features, and the fact that the distinction between normal and abnormal brain development is not defined by an unambiguous cutoff point.

Still, brain MR imaging plays a substantial role in clinical decision making regarding the management of children with spina bifida [9, 10, 12]. On the one hand, the discussion on selective treatment of severely affected newborn infants is still ongoing [13]. On the other hand, fetal imaging and prenatal surgery are becoming more important every day. Recently, a randomized control trial showed important improvement of hindbrain herniation following prenatal surgery for spina bifida [14]. However, the assessment of Chiari II malformation may be even more complicated in prenatal MR imaging. A discrepancy of 41 % was seen in judgment of the degree of hindbrain herniation in prenatal MR imaging studies [15]. When choices have to be made about pre- and postnatal treatment options, morphometric analyses may improve the assessment of severity of Chiari II malformation on MR images in clinical and research settings. Measurements of the cerebellum, brainstem, and posterior fossa may give quantitative information about the extent of the malformation and may provide objective cutoff points between normal and abnormal brain development. A few morphometric studies on Chiari II malformation have been reported [16–21]. These studies generally focused on the small posterior fossa and the degree of cerebellar herniation in the midsagittal plane, but not on dimensions in the axial or coronal plane. Interobserver reliability and diagnostic performance of the morphometric measures are hardly addressed in the literature.

Therefore, we investigated the interobserver reliability and diagnostic performance of morphometric measures of the cerebellum, brainstem, and posterior fossa, not only in the midsagittal plane but also in the axial and coronal plane, to select appropriate measures for the MR assessment of Chiari II malformation.

## Materials and methods

### Patients

Brain MR images of 79 children [mean age 10.6 (SD 3.2; range, 6–16) years] were evaluated. Of these children, 43 children had spinal dysraphism (26 with open spinal dysraphism and 17 with closed spinal dysraphism [22]). The majority of these children (*n* = 36) were recruited at the outpatient clinics of Pediatric Neurology of the Radboud University Nijmegen Medical Centre (RUNMC) as part of a prospective research program dedicated to the outcome and prognosis of spina bifida. MR images of the remaining seven children were obtained retrospectively from the archives of the Department of Radiology of the RUNMC, from which we also obtained brain MR images of 36 children without spinal dysraphism. Although MR imaging in these 36 children was performed with suspicion of or to rule out cerebral pathology, the MR images had been assessed as normal by an independent radiologist in a clinical setting before the start of the study. All 79 children were reassessed for Chiari II malformation using the criteria: cerebellar herniation on a sagittal MR image and the presence of open spinal dysraphism. Consequently, the study population consisted of three diagnostic groups: 23 children with spinal dysraphism and Chiari II malformation [SDCM+ group; mean age 11.4 (SD 2.9; range, 6–16) years], 20 children with spinal dysraphism, but without Chiari II malformation [SDCM− group; mean age 10.9 (SD 3.1; range, 7–16) years], and 36 children without spinal dysraphism or cerebral pathology [reference group; mean age 9.9 (SD 3.2; range, 6–16) years].

### MR imaging

All MR images were acquired using a 1.5-T MR imaging unit (Siemens Avanto; Siemens Medical Solutions, Erlangen, Germany) with a standard head coil. MR imaging in the 36 children who were part of the prospective research program consisted of T1-weigthed images in the sagittal plane and T2-weigthed images in the axial and coronal plane. The retrospectively obtained MR images were acquired using comparable sequences. For different reasons, MR images were not acquired in three planes for all 79 children. Images in the sagittal plane were available for 69 children (21 in the SDCM+ group, 20 in the SDCM− group, and 28 in the reference group), images in the axial plane for 58 children (19 in the SDCM+ group, 13 in the SDCM− group, and 26 in the reference group), and images in the coronal plane for 51 children (18 in the SDCM+ group, 19 in the SDCM− group, and 14 in the reference group).

The Regional Committee on Research involving Human Subjects approved the study protocol. Prior to inclusion in the study, written informed consent was obtained from the parents of all 36 children and all children above 12 years of age taking part in the prospective research program.

### Image analysis

All MR images were blinded for demographic and diagnostic information. The MR images of the three diagnostic groups were mixed and arranged by plane into three data sets: a sagittal set, an axial set, and a coronal set. These three data sets were reviewed independently by three observers: a junior pediatric neurologist (N.G.) with 6 years of experience in reviewing pediatric brain MR images, a senior pediatric neurologist (R.A.M.), and a senior neuroradiologist (T.V.), both with more than 20 years of experience in reviewing pediatric brain MR images. The images were available on compacts disks and were reviewed on an Agfa workstation or on a personal computer using Agfa software (Impax Client, release 4.5).

The MR images were reviewed for 13 sagittal, 4 axial, and 4 coronal morphometric measures (Table 1). Most of the measures in the sagittal plane were selected from the literature. The measures in the axial and coronal plane were defined by the authors to appraise the width of the cerebellum, the degree of wrapping of the cerebellar hemispheres around the brainstem, and the degree of upward tentorial herniation of the cerebellar hemispheres.

**Table 1:** Morphometric measures of Chiari II malformation

Measure	Definition	Reference
Sagittal plane^a^		
Foramen magnum diameter	Distance between basion and opisthion	Aboulezz et al. [16]
Vermis level	Distance perpendicular from the line between basion and opisthion, to the most caudal extent of the vermis^b^	Modified from Barkovich et al. [29]
Tonsil level	Distance perpendicular from the line between basion and opisthion, to the most caudal extent of the tonsil^b^	Barkovich et al. [29]
Kinking level	Distance perpendicular from the line between basion and opisthion, to the most caudal and dorsal border of the kink	Modified from Barkovich et al. [29]
Fourth ventricle level	Distance perpendicular from the line between basion and opisthion, to the fastigium of the fourth ventricle	Modified from Barkovich et al. [29]
Cerebellar height	Distance from the most rostral point of the cerebellum to the most caudal extent of the cerebellum	Salman et al. [21]
Vermis length	Sagittal distance from the fastigium of the fourth ventricle to the most dorsal part of the vermis	Salman et al. [21]
Medulla length	Distance from the superior pontine notch to the cervicomedullary junction	Nishikawa et al. [30]
Pons length	Distance from the superior pontine notch to the inferior pontine notch	Tsai et al. [20]
Pons thickness	Distance from the ventral side of the pons to the dorsal side of the medulla, perpendicular to the line representing the pons length, at the middle of this line	Modified from Barkovich. [1]
Mamillopontine distance	Distance from the inferior border of the mamillary body to the superior bulge of the pons	El Gammal et al. [28]
Tentorial length^c^	Distance from the tentorial insertion at the cortex of the skull to the edge of the tentorium	
Cisterna magna width^c^	Distance from the opisthion to the vermis perpendicular to the line between opisthion and tentorial insertion	
Axial plane		
Cerebellar width^c^	Distance from the most lateral border of the right hemisphere to the most lateral border of the left hemisphere, perpendicular to the midsagittal line, independent of the MR image slice level	
Hemispheral length^c^ (left and right)	In the same slice as the Cerebellar width, distance from the most rostral border of the cerebellar hemisphere to the most posterior border of the cerebellar hemisphere, parallel to the midsagittal line	
Vermis length^c^	Maximal distance from anterior vermis border to posterior vermis border, independent of the MR image slice level	
Coronal plane		
Cerebellar width^c^	In the slice just posterior to the fourth ventricle, distance from the most lateral border of the right hemisphere to the most lateral border of the left hemisphere, perpendicular to the midsagittal line	
Hemispheral height^c^ (left and right)	In the slice just posterior to the fourth ventricle, distance from the most cranial border of the cerebellar hemisphere to most caudal border of the cerebellar hemisphere, parallel to the midsagittal line	
Vermis length^c^	Distance from the most rostral vermis border to the most caudal vermis border, independent of the MR image slice level	

^a^All sagittal measurements were performed in the midsagittal plane

^b^If above the foramen magnum, provided with a positive sign, and if below the foramen magnum, with a negative sign

^c^Measure introduced in the present study

First, the feasibility of the protocol was evaluated in a pilot study (*n* = 10), resulting in the final set of measures with their definitions. Measures were assessed to the nearest decimal of a millimeter. If an observer could not identify a landmark or could not assess the measure for other reasons, the measurement was classified as “indeterminable.”

### Statistical analysis

For each measure, the indeterminable measurements were tallied up per observer to assess the feasibility of each measure. If at least two observers considered a measure to be indeterminable in more than 5 % of the MR images, the measure was qualified as unfeasible and subsequently excluded from the further analyses.

The interobserver agreement of the feasible measures was quantified by the agreement index (AI), defined as AI = 1 − RRE, where RRE denotes the relative random measurement error expressed as the pooled coefficient of variation across patients of the observations made by the three observers. This AI can be seen as an extension to more than two observers of the AI defined for two observations per patient [23, 24]. The relative random measurement error was used instead of the absolute random measurement error in order to compare measures among each other. An AI ≥ 0.90 was considered to indicate reliable interobserver agreement. Using this method, the overall interobserver agreement, the interobserver agreement between pairs of observers, and the interobserver agreement per diagnostic group were calculated.

The reliable measures were also analyzed for diagnostic performance regarding Chiari II malformation. Initially, the measurements of observer A were used for this purpose. Differences between the three diagnostic groups were analyzed with the Kruskal–Wallis test. Using the diagnosis of Chiari II malformation (defined as cerebellar herniation on a sagittal MR image and presence of open spinal dysraphism) as the reference standard, a receiver operating characteristic (ROC) curve was constructed for each measure. The area under the ROC curve (AUC) and its 95 % confidence interval (CI) were calculated to assess the diagnostic performance. The cutoff value with the optimal sensitivity and specificity was ascertained from the curve. Subsequently, the consistency of the measures with a high diagnostic performance (AUC > 0.90) was assessed using the measurements of the other two observers. All statistical analyses were performed using SPSS software version 14.0.1.

## Results

### Reliability

Most measures turned out to be feasible, except for *fourth ventricle level* in the sagittal plane and *vermis length* in the axial and coronal planes. These three measures were excluded from the further interobserver agreement and diagnostic performance analyses.

The interobserver agreement of the remaining measures is presented in Table 2. For most measures, the interobserver agreement was reliable (AI ≥ 0.9), both overall and per diagnostic group. In general, the agreement was slightly weaker in the SDCM+ group than in the other diagnostic groups, but this difference was only meaningful for *tentorial length*. The agreement was very poor for *vermis level*, *tonsil level*, and *cisterna magna width*. The interobserver agreement for pairs of observers showed that the poor agreement for *cisterna magna width* and *tonsil level* were not observer dependent. The poor agreement for *vermis level*, however, was observer dependent (Table 3). For all other measures, pairwise agreement did not differ among pairs of observers.

**Table 2:** Agreement indexes (calculated as 1 − RRE; for further details, see “Materials and methods”) of morphometric measures overall and per diagnostic group

Measure	Overall	SDCM+	SDCM−	Reference group
Sagittal plane				
Foramen magnum diameter	*0.93* ^a^	0.91	0.97	0.94
Vermis level	0.06	−0.25	0.25	0.26
Tonsil level	0.20	0.38	0.41	0.36
Kinking level	*0.92*	0.93	–^b^	–^b^
Cerebellar height	*0.92*	0.87	0.97	0.98
Vermis length	*0.93*	0.93	0.92	0.94
Medulla length	*0.92*	0.90	0.93	0.93
Pons length	*0.94*	0.91	0.96	0.98
Pons thickness	*0.95*	0.93	0.97	0.95
Mamillopontine distance	*0.91*	0.94	0.90	0.89
Tentorial length	0.88	0.76	0.92	0.92
Cisterna magna width	0.40	−1.57	0.48	0.54
Axial plane				
Cerebellar width	*0.93*	0.86	0.98	0.98
Hemispheral length left	0.88	0.87	0.87	0.91
Hemispheral length right	0.89	0.89	0.89	0.90
Coronal plane				
Cerebellar width	*0.98*	0.98	0.99	0.99
Hemispheral height left	*0.91*	0.89	0.92	0.91
Hemispheral height right	*0.90*	0.91	0.90	0.92

*SDCM+* spinal dysraphism with Chiari II malformation, *SDCM*− spinal dysraphism without Chiari II malformation

^a^Values ≥ 0.90 are indicated in italic.

^b^Kinking was not present in the SDCM− group and in the reference group

**Table 3:** Agreement indexes (calculated as 1 − RRE; for further details, see “Materials and methods”) of the three measures with poor interobserver agreement, overall and by observer pair

Measure	Overall	Observer pairs		
		A–B	A–C	B–C
Vermis level	0.06	0.69	−0.12	−0.19
Tonsil level	0.20	0.33	0.15	0.10
Cisterna magna width	0.40	0.39	0.41	0.39

*A* observer A, *B* observer B, *C* observer C

### Diagnostic performance

In the sagittal and axial plane, all but one measure differed statistically significantly between the SDCM+ group and the other two diagnostic groups (Table 4). In the coronal plane, only *cerebellar width* was statistically significantly smaller in the SDCM+ group than in the other two groups. No differences were present between the SDCM− group and the reference group.

**Table 4:** Measurements (mean values in cm) by diagnostic group (data obtained from observer A)

Measure	SDCM+		SDCM−	Reference group		*P* value^a^
Sagittal plane						
Foramen magnum diameter	4.46	(4.35[16])^b^	3.62	3.64	(3.68[16])	<0.0001
Kinking level	−3.56		–^c^	–^c^		
Cerebellar height	6.94	(6.8[21])	5.84	5.68	(5.5[21])	<0.0001
Vermis length	3.60	(3.7[21])	3.00	2.91	(3.0[21])	<0.0001
Medulla length	6.03		5.55	5.41		<0.05
Pons length	3.27	(2.9[20])	2.59	2.56	(2.7[20])	<0.0001
Pons thickness	1.87		2.24	2.21		<0.0001
Mamillopontine distance	1.34		0.74	0.72		<0.0001
Axial plane						
Cerebellar width	8.01		10.22	10.30		<0.0001
Hemispheral length left	5.18		5.86	5.73		0.06
Hemispheral length right	5.09		5.71	5.76		<0.001
Coronal plane						
Cerebellar width	8.55		9.98	9.91		<0.001
Hemispheral height left	5.61		5.46	5.42		0.46
Hemispheral height right	5.46		5.40	5.50		0.77

*SDCM+* spinal dysraphism with Chiari II malformation, *SDCM*− spinal dysraphism without Chiari II malformation

^a^ *P* values for differences between the three diagnostic groups based on the Kruskal–Wallis test

^b^Values between brackets are reference values from the literature

^c^Kinking was not present in the SDCM− group and in the reference group

**Fig. 1:**
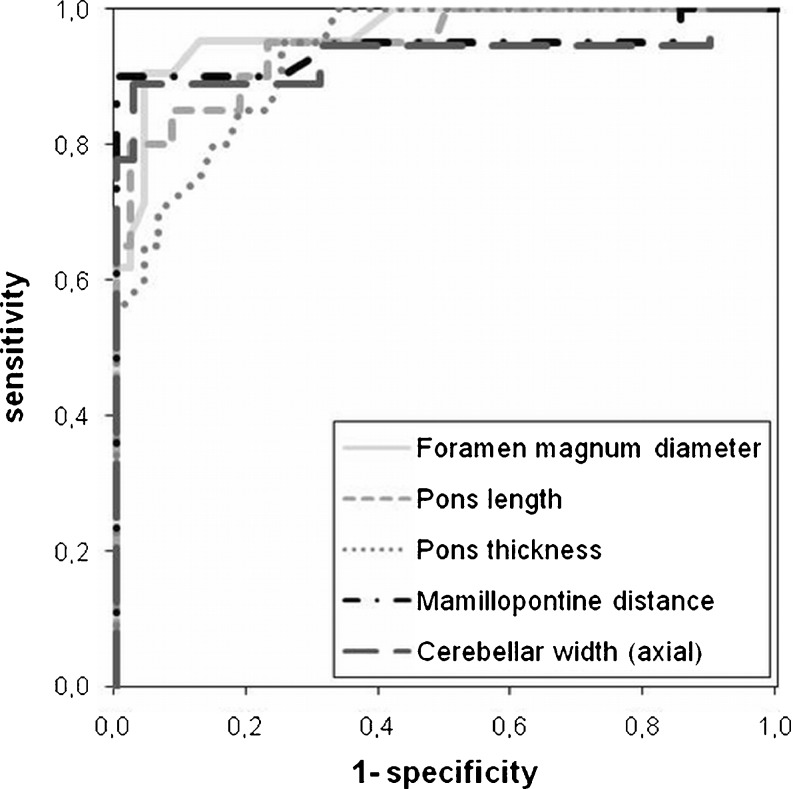
Receiver operating characteristic curves for measures with a good diagnostic performance (AUC > 0.90). See Table 5 for further details

**Fig. 2:**
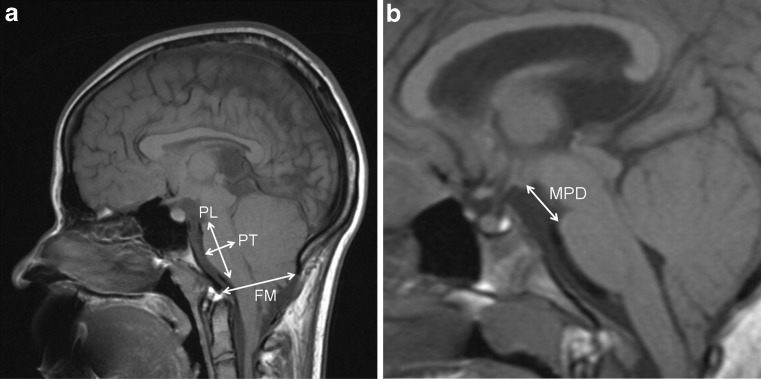
**a** Sagittal T1-weighted brain MR image of a 16-year-old child with open spinal dysraphism and Chiari II malformation. The *arrows* indicate foramen magnum diameter (*FM*), pons length (*PL*), and pons thickness (*PT*); **b** sagittal T1-weighted brain MR image of a 8-year-old child with open spinal dysraphism and Chiari II malformation. The arrow indicates mamillopontine distance (*MPD*)

**Fig. 3:**
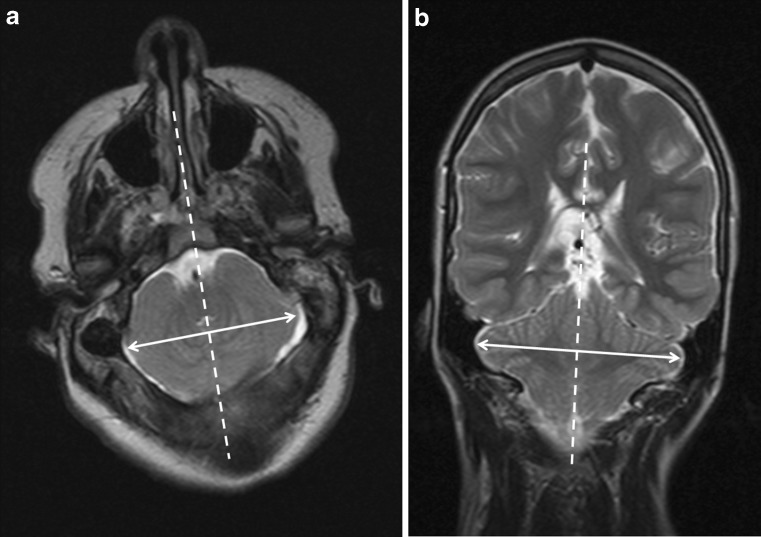
**a** Axial T2-weighted brain MR image of a 16-year-old child with open spinal dysraphism and Chiari II malformation. The *arrow* indicates axial cerebellar width; **b** coronal T2-weighted brain MR image of a 13-year-old child with open spinal dysraphism and Chiari II malformation. The *arrow* indicates coronal cerebellar width

The diagnostic performance of the measures based on the data from observer A is presented in Table 5 and illustrated by ROC curves in Fig. 1. The AUC was substantial (>0.90) for five measures: *foramen magnum diameter*, *pons length*, *pons thickness*, and *mamillopontine distance* in the sagittal plane (Fig. 2), and *cerebellar width* in the axial plane (Fig. 3), but sensitivity or specificity were not all that high for *pons length* and *pons thickness*. Consistency of the performance of these five measures was evaluated using the measurement values of observers B and C (Table 6). In this analysis, only *mamillopontine distance* and *cerebellar width* maintained their excellent diagnostic performance. Despite the high sensitivity and specificity in the primary analysis, *foramen magnum diameter* failed to the consistency test.

**Table 5:** Results of ROC analyses showing the diagnostic performance of Chiari II malformation measures (data obtained from observer A)

Measure	AUC	95 % CI	Sensitivity	Specificity	Cutoff value (cm)
Sagittal plane					
Foramen magnum diameter	0.97	0.93–1.01	0.90	0.96	3.94
Cerebellar height	0.87	0.76–0.98	0.85	0.90	6.31
Vermis length	0.88	0.76–0.99	0.88	0.88	3.19
Medulla length	0.72	0.56–0.87	0.50	0.94	6.07
Pons length	0.95	0.89–1.01	0.80	0.98	2.96
Pons thickness	0.93	0.88–0.99	0.95	0.75	2.14
Mamillopontine distance	0.94	0.86–1.03	0.90	1.00	1.05
Axial plane					
Cerebellar width	0.93	0.83–1.03	0.89	0.97	9.57
Hemispheral length left	0.68	0.51–0.85	0.53	0.90	5.22
Hemispheral length right	0.82	0.70–0.95	0.71	0.90	5.30
Coronal plane					
Cerebellar width	0.82	0.68–0.97	0.76	0.88	9.43
Hemispheral height left	0.52	0.34–0.69	0.18	0.94	6.04
Hemispheral height right	0.61	0.42–0.79	0.53	0.81	5.80

*AUC* area under the receiver operating characteristic (ROC) curve

**Table 6:** Consistency [tested by applying the results of the ROC analysis (see Table 5) to the data obtained from observer B and C] of the measures with the best diagnostic performance in the ROC analyses

Measure	Sensitivity	Specificity	Cutoff value (cm)
Foramen magnum diameter	0.69	0.79	3.94
Pons length	0.68	0.96	2.96
Pons thickness	0.93	0.59	2.14
Mamillopontine distance	0.84	0.97	1.05
Cerebellar width (axial plane)	0.89	0.92	9.57

## Discussion

On brain MR images, Chiari II malformation is generally evaluated based on a constellation of morphological characteristics in the midsagittal plane. The current study provides quantitative measures that may provide information about the extent or severity of Chiari II malformation. The measures *mamillopontine distance* and *cerebellar width* seem to be highly specific and sensitive for assessing Chiari II malformation.

In the present study, most measures turned out to be reliable, both overall and per diagnostic group. The literature provides some morphometric studies of Chiari II malformation [16–21, 25], but only the study of Salman et al. [21] deals with interobserver agreement of several measures. As far as the same measures were studied, our results agree with the previous findings. The additional value of our study is that we investigated measures in three planes and in different diagnostic groups. The interobserver agreement in the Chiari II malformation group was slightly lower than in the unaffected groups. This may be due to anatomical distortions, which may hamper precise identification of landmarks. However, this did not affect reliability to a large extent.

Unreliable measures in the present study were predominantly complex measures, depending on reference lines, which are susceptible to differences in interpretation as well. For example, the disagreement found for *foramen magnum diameter* will have contributed to the disagreement for the measures that depend on it, such as *vermis level*.

The unreliability of *vermis level* and *tonsil level* was remarkable. Blurred boundaries in a crowed posterior fossa and upper cervical spinal canal may have hampered precise delineation of the tonsils and vermis. Consequently, these structures could not be distinguished precisely. On the other hand, the disagreement for *vermis level* may also be observer dependent, as two of the three observers moderately agreed on *vermis level*, whereas these two observers systematically disagreed with the third observer (Table 3). To elucidate this, we performed a post hoc analysis using the most caudal extent of cerebellar tissue (vermis or tonsil) as a variable. As this derivative measure also failed to be reliable (AI = 0.29), however, observer dependency seems to play a minor role. In contrast, Salman et al. [21] presented a comparable measure “herniation distance” as reliable, but they used other statistical methods in a smaller sample size. Although cerebellar herniation remains a key feature of Chiari II malformation and its morphological appearance can reliably be judged on MR images (see part 1), the present study shows that measuring the degree of cerebellar herniation can be unreliable.

The majority of the reliable measures differed statistically significantly between children with Chiari II malformation and unaffected children (Table 4). These differences are in accordance with the morphogenesis of Chiari II malformation. Increased *cerebellar height* and *vermis length* and decreased *cerebellar width* support the hypothesis of a small posterior fossa [3] with squeezing of the vermis and enlargement of the midsaggital vermis area [21]. An increased *mamillopontine distance* results from caudal displacement of the brainstem and pons. For a few measures, reference values have been reported in the literature (Table 4). Our values for *foramen magnum diameter* corresponded well with the values reported by Aboulezz et al. [16] and our values for *cerebellar height* and *vermis length* with the values reported by Salman et al. [21]. The *pons length* in affected children in our study was longer than the *pons length* reported by Tsai et al. [20]. A different identification of the inferior pontine notch and a different age range of the investigated populations might explain this difference.

The substantial differences in the measurement values between affected and unaffected children warrant the search for cutoff points. The ROC analyses showed reasonably accurate cutoff points for more than half of the reliable measures (Table 5), but only two measures, *mamillopontine distance* and *cerebellar width*, showed consistent diagnostic performance. Some caution is justified, however. From the ROC analyses, very precise cutoff points were calculated, but this amount of precision will not be feasible in clinical practice.

Clinicians should be aware of the imprecise judgment of the degree of cerebellar herniation in the midsagittal plane. The reliable measures presented are more suitable to assess the morphological distortions. They appraise the cerebellum and brainstem not only in the midsagittal plane but also in the axial and coronal plane. Since measures differ substantially between affected and unaffected children, they are considered to be of diagnostic value. *Cerebellar width* provides an indication of the size of the posterior fossa, and *cerebellar height* and *vermis length* reflect the enlarged vermis area. *Mamillopontine distance*, *pons length*, and *medulla length* provide quantifications of downward displacement and stretching of the brainstem. Although *hemispheral length* and *hemispheral height* were reliable measures, they did not differ substantially between affected and unaffected children and thus failed to provide objective cutoff values for wrapping of the cerebellar hemispheres around the brainstem and upward tentorial herniation, respectively. The reliable measures might be suitable to assess severity of clinical signs and symptoms. However, the association between measurements and severity of Chiari II malformation is a matter of further study.

The results of this study may have implications for prenatal surgery for spina bifida as well. Intrauterine spina bifida repair appears to reverse the degree of hindbrain herniation [14, 26, 27]. The currently used scoring system might be imprecise, as it is based on the degree of vermis herniation and the position of the fourth ventricle. The present study provides reliable measures, which may be more suitable to objectively evaluate the effect of prenatal surgery on Chiari II malformation in three dimensions. However, the results may not simply be transformed to prenatal imaging, since unshunted hydrocephalus might have an effect on the measures in the prenatal setting. In particular, this may be relevant for *mamillopontine distance*, as this distance may decrease as a result of raised intracranial pressure [28]. The effect of hydrocephalus may have less influence on most other measures. However, additional evaluation of the measures in a prenatal setting is recommended.

The study also had some limitations. Due to its partly retrospective design, the study population comprised a heterogeneous set of MR images. Furthermore, the reference standard used in the ROC analyses might be questionable. However, a better reference standard is currently not available. Finally, we could not take into account a possible age effect even though brain dimensions change in a growing child. However, Salman et al. [21] showed that MR measurements of the posterior fossa did not correlate with age in children with Chiari II malformation. In the present study, the strong differences between affected and unaffected children seem to outweigh the influence of age.

In conclusion, using morphometric measures represent a reliable and feasible method to quantify the morphological distortions of Chiari II malformation on MR images. These measures are easily used on standard MR images without the need of specific software. They appraise different parts of the cerebellum, brainstem, and posterior fossa providing quantitative information about the extent of Chiari II malformation in three dimensions. The measures may have added value in assessment of severity of Chiari II malformation in clinical decision making as well as in research settings, such as studies on the effect of prenatal surgery for spina bifida. The excellent diagnostic performance of *mamillopontine distance* and *cerebellar width* makes these measures particularly helpful in cases in which the diagnosis of Chiari II malformation is ambiguous.

## References

[1] Barkovich AJ, Barkovich AJ (2005). Congenital malformations of the brain and skull. Pediatric neuroimaging.

[2] Chiari H (1891). Ueber Veränderungen des Kleinhirns infolge von Hydrocephalie des Grosshirns. Deut Med Wochenschr.

[3] McLone DG, Knepper PA (1989). The cause of Chiari II malformation: a unified theory. Pediatr Neurosci.

[4] Stevenson KL (2004). Chiari type II malformation: past, present, and future. Neurosurg Focus.

[5] McLone DG (1992). Continuing concepts in the management of spina bifida. Pediatr Neurosurg.

[6] Oakeshott P, Hunt GM (2003). Long-term outcome in open spina bifida. Br J Gen Pract.

[7] Wolpert SM, Anderson M, Scott RM, Kwan ES, Runge VM (1987). Chiari II malformation: MR imaging evaluation. AJR Am J Roentgenol.

[8] El Gammal T, Mark EK, Brooks BS (1988). MR imaging of Chiari II malformation. AJR Am J Roentgenol.

[9] Just M, Schwarz M, Ludwig B, Ermert J, Thelen M (1990). Cerebral and spinal MR-findings in patients with postrepair myelomeningocele. Pediatr Radiol.

[10] Kawamura T, Morioka T, Nishio S, Mihara F, Fukui M (2001). Cerebral abnormalities in lumbosacral neural tube closure defect: MR imaging evaluation. Childs Nerv Syst.

[11] Miller E, Widjaja E, Blaser S, Dennis M, Raybaud C (2008). The old and the new: supratentorial MR findings in Chiari II malformation. Childs Nerv Syst.

[12] Mitchell LE, Adzick NS, Melchionne J, Pasquariello PS, Sutton LN, Whitehead AS (2004). Spina bifida. Lancet.

[13] Barry S (2010). Quality of life and myelomeningocele: an ethical and evidence-based analysis of the Groningen Protocol. Pediatr Neurosurg.

[14] Adzick NS, Thom EA, Spong CY (2011). A randomized trial of prenatal versus postnatal repair of myelomeningocele. N Engl J Med.

[15] Mangels KJ, Tulipan N, Tsao LY, Alarcon J, Bruner JP (2000). Fetal MRI in the evaluation of intrauterine myelomeningocele. Pediatr Neurosurg.

[16] Aboulezz AO, Sartor K, Geyer CA, Gado MH (1985). Position of cerebellar tonsils in the normal population and in patients with Chiari malformation: a quantitative approach with MR imaging. J Comput Assist Tomogr.

[17] Wolpert SM, Scott RM, Platenberg C, Runge VM (1988). The clinical significance of hindbrain herniation and deformity as shown on MR images of patients with Chiari II malformation. AJNR Am J Neuroradiol.

[18] Curnes JT, Oakes WJ, Boyko OB (1989). MR imaging of hindbrain deformity in Chiari II patients with and without symptoms of brainstem compression. AJNR Am J Neuroradiol.

[19] Ruge JR, Masciopinto J, Storrs BB, McLone DG (1992). Anatomical progression of the Chiari II malformation. Childs Nerv Syst.

[20] Tsai T, Bookstein FL, Levey E, Kinsman SL (2002). Chiari-II malformation: a biometric analysis. Eur J Pediatr Surg.

[21] Salman MS, Blaser SE, Sharpe JA, Dennis M (2006). Cerebellar vermis morphology in children with spina bifida and Chiari type II malformation. Childs Nerv Syst.

[22] Tortori-Donati P, Rossi A, Cama A (2000). Spinal dysraphism: a review of neuroradiological features with embryological correlations and proposal for a new classification. Neuroradiology.

[23] Filippi M, Horsfield MA, Bressi S (1995). Intra- and inter-observer agreement of brain MRI lesion volume measurements in multiple sclerosis. A comparison of techniques. Brain.

[24] Joe BN, Fukui MB, Meltzer CC (1999). Brain tumor volume measurement: comparison of manual and semiautomated methods. Radiology.

[25] Grant RA, Heuer GG, Carrion GM (2011). Morphometric analysis of posterior fossa after in utero myelomeningocele repair. J Neurosurg Pediatr.

[26] Tulipan N, Hernanz-Schulman M, Bruner JP (1998). Reduced hindbrain herniation after intrauterine myelomeningocele repair: a report of four cases. Pediatr Neurosurg.

[27] Tulipan N, Hernanz-Schulman M, Lowe LH, Bruner JP (1999). Intrauterine myelomeningocele repair reverses preexisting hindbrain herniation. Pediatr Neurosurg.

[28] El Gammal T, Allen MB, Brooks BS, Mark EK (1987). MR evaluation of hydrocephalus. AJR Am J Roentgenol.

[29] Barkovich AJ, Wippold FJ, Sherman JL, Citrin CM (1986). Significance of cerebellar tonsillar position on MR. AJNR Am J Neuroradiol.

[30] Nishikawa M, Sakamoto H, Hakuba A, Nakanishi N, Inoue Y (1997). Pathogenesis of Chiari malformation: a morphometric study of the posterior cranial fossa. J Neurosurg.

